# Morphological Characterization of Two Light Italian Turkey Breeds

**DOI:** 10.3390/ani12050571

**Published:** 2022-02-24

**Authors:** Stefano Paolo Marelli, Luisa Zaniboni, Maria Giuseppina Strillacci, Manuela Madeddu, Silvia Cerolini

**Affiliations:** Dipartimento di Medicina Veterinaria e Scienze Animali, Università Degli Studi di Milano, Via Dell’Università 6, 26900 Lodi, Italy; stefano.marelli@unimi.it (S.P.M.); maria.strillacci@unimi.it (M.G.S.); manuela.madeddu@unimi.it (M.M.); silvia.cerolini@unimi.it (S.C.)

**Keywords:** turkey breeds, morphometry, biodiversity, traditional products, Brianzolo, Nero d’Italia

## Abstract

**Simple Summary:**

Genetic resources on turkeys are scarce, considering both domestic and wild birds. Smaller, dark-colored Italian heritage breeds such as Brianzolo and Nero d’Italia have been selected for centuries in outskirt areas due to their coping ability in poor environments and high brooding ability. However, they are in danger of extinction. EC Council Regulations on organic farming strongly recommend using local genetic resources and conserving biodiversity. Phenotypical characterization is a basic step in biodiversity conservation; therefore, in this article, we present morphometric and colorimetric analysis results of 46 birds belonging to each of the considered breeds. We considered 18 parameters: morphometries, morphological indexes, linear evaluation, and colorimetric indexes as characterizing factors in breeds differentiation. Differences in weight with clear sexual dimorphism were recorded. Massiveness indexes were in favor of the slightly more compact Brianzolo breed. The extremely fair skin and dark shank of the Nero d’Italia turkey were revealed by colorimetric analysis. The effectiveness of morphological analysis in breeds differentiation, useful data in heritage breeds conservation, and characterization were considered control parameters in classifying animal products.

**Abstract:**

We aimed to investigate the variability within turkeys’ phenotypical traits in two Italian heritage breeds: Brianzolo (BRZ) and Nero d’Italia (NIT), as analyzed through morphometry, morphometrical indexes, linear scoring, and colorimetric indexes. A total of 92 birds were measured, weighed, and scored (46 NIT: M/F = 19/27; 46 BRZ: M/F = 19/27). Live weight (LW), total body length (BL, excluding feathers), keel length (KL), chest circumference (BC), wingspan (WS), shank length (SL), shank diameter (SD), and shank circumference (SC). Massiveness (MASS), stockiness (STOCK), and long-leggedness (LLEG) indexes were also calculated. The body condition score (BCS) applied a linear evaluation to nutritional status and muscular development. Colorimetric indexes (L*, a*, b*) were recorded, sampling skin and shank. Data were analyzed using GLM procedures and PCA. NIT was the heaviest breed (4.89 vs. 4.07 kg; *p* ≤ 0.05). In both breeds, sexual dimorphism was visible in the LW trait with males (M) weighing significantly heavier than females (F) (*p* ≤ 0.05). NIT birds recorded the highest BL values: 58.44 vs. 57.15 cm (*p* ≤ 0.05). MASS was higher in NIT (8.26 vs. 7.0; *p* ≤ 0.05), and STOCK was higher in BRZ (82.62 vs. 85.37; *p* ≤ 0.05). Colorimetric indexes revealed significant differences in skin lightness (L*) and redness (a*). For shank color, the breed significantly affected differences in the indexes. This study characterizes these breeds at high risk of genetic erosion and extinction, which will help the morphological standardization of birds and the enhancement of genetic variability

## 1. Introduction

Italy is characterized by high genetic variability in every domesticated species due to its high environmental differentiation. Furthermore, the constant migration of humans and animals through the Italian territory favored this diversity. In previous decades, the abandonment of remote mountain areas for peri-urban zones, where the population is mainly concentrated, particularly in highly industrialized planes, strongly affected the population size of heritage breeds [1]. Autochthonous poultry breeds result from many generations of adaptation to particular environments, cultural needs, and consumer preferences [2]. After the Second World War, agricultural industrialization and the development of commercial hybrid strains led to a progressive reduction in local population numbers and potential extinction in many poultry species [1,3]. The commercial goal of selecting effective and productive traits in birds with balanced industrial diets in controlled environments caused them to lose their adaptability to free-range systems and low-input diets based on pastures and motor activity. Furthermore, this substitution with commercial strains involved the entire world’s poultry production and displaced autochthonous populations [1,3]. Biological information on heritage turkey breeds and populations are important aspects to consider in conservation and selection programs due to differences within local turkey breeds and between local breeds and commercial strains. Furthermore, local breeds are an irreplaceable genetic reservoir for the implementing commercial strain characteristics [3,4]. The genetic variability in highly productive species such as poultry has been reduced by selective breeding. Therefore, the conservation of traditional turkey breeds is a helpful action that provides effective genetic variability and unique traits [3,5]. Some objectives include supporting the conservation of endangered domestic animals by using these breeds in extensive production systems, protecting the cultural heritage behind local breeds as an expression of human–environment interaction, and reducing genetic variability loss for productive and health-related reasons [1]. The genetic diversity of turkeys (*Meleagris gallopavo*) is threatened in the wild, where subspecies integrity is at risk [6] and in meat production systems [4]. Product characterization and differentiation may be an effective strategy for supplying economic support to pure breed conservation strategies and planning. In addition, the European Union’s regulations for organic farming and the labeling of organic products stress the importance of slow-growing local breeds adapted to welfare-friendly systems characterized by outdoor space availability (Council Regulation (EC) No 834/2007; European Parliament and Council Regulation (EU) 2018/848).

Phenotypical evaluation plays a pivotal role in animal conservation genetic resources and molecular genetic investigations [3,4,5,7,8]. Morphological traits are easily observed and successfully used in slow-growing pure breed turkey promotion policies [9,10,11,12,13]. Furthermore, product quality traits directly influence consumer choices, such as the colorimetric evaluation of skin color, which supplies objective discriminants in the marketing of traditional poultry products [14]. The conservation of turkey heritage breeds can be oriented to niche high-quality products with extensive production systems [15].

Nero d’Italia (NIT) and Brianzolo (BRZ) (Table 1) are very ancient breeds originating from Northern Italy; their development is strictly related to historical events and human migration across Northern Italian regions [2,16].

We aimed to investigate variability within turkeys’ phenotypical traits as analyzed through morphometry, morphometrical indexes, linear scoring, and colorimetric indexes. According to FAO protocols, these methods supply objective information and data useful for characterizing traditional breeds at risk of extinction for conservation purposes [5]. Furthermore, the differentiation in some colorimetric traits related to heritage breed products promotes slow-growing, low-input production systems. Objective data based on scientific and productive parameters have been implemented for updating official breed standards included in the National Herd Book of autochthonous poultry breeds (DG DISR n. 0019536, 1 October 2014).

## 2. Materials and Methods

### 2.1. Animals

A total of 92 adult birds (age ≥ 20 weeks) from two traditional Italian turkey breeds, BRZ and NIT, were measured, weighed, and scored at the Poultry Unit of the University of Milan’s Animal Production Center (Lodi, Italy), where the official breeding flocks of the two breeds were reared (Ministry of Agricultural, Food, Forestry, and Tourism Policies: PSRN 2014–2020). The sample size was 46 BRZ birds (17 toms + 29 hens) and 46 NIT birds (17 toms + 29 hens). Turkeys were reared in indoor pens (3 × 2 m^2^; 1 m + 2F) and fed ad libitum (2800 Kcal ME/kg, CP 17.5%, EE 3.7%). Drinking water was ad libitum available to the birds.

### 2.2. Phenotypic Data Collections

Phenotypic data were recorded during routine weight, and nutritional status controls according to health authority protocols and animal welfare legislation to ensure birds’ maximum health and welfare conditions. Phenotypic data are grouped into three different sections: morphometrical, linear evaluation, and colorimetric measures.

The following morphometrical measures were recorded: live weight (LW), total body length (tip of the beak to the tip of the tail excluded feathers; BL), keel length (KL), chest circumference (measured at the tip of the hind chest; BC), wingspan (tip of right to tip of the left wing, wings fully stretched out; WS), shank length (SL), shank diameter (SD), and shank circumference (SC), according to FAO guidelines for the phenotypic characterization of animal genetic resources [5,17]. Shank length and girth were measured from the left shank. Used tools: weight—XPR32001L Balance (Keller Toledo^®^, Milan, Italy); length, circumference—Seca201 soft measuring tape (Seca^®^, Hamburg, Germany), diameters—Juning Caliper 150 mm/0.1 mm (Juning^®^, Changzhou, China).

Massiveness (MAS; body weight/body length ratio, kg/cm), stockiness (STOCK; chest circumference/body length ratio, cm/cm) [18], and long-leggedness (LLEG; shank length/body length ratio, cm/cm) [19] were calculated.

The body condition score (BCS) applies a linear evaluation to nutritional status and muscular development: 0 = protruding keel bone and depressed contour to breast muscles; 1 = prominent keel bone with a poorly developed breast; 2 = less prominent keel bone and moderate breast muscle development; 3 = plump breast muscles that provide a smooth contour with the keel [20].

Colorimetric data (L*—lightness, a*—redness, b*—yellowness) were recorded on the skin, apterium area under the left wing, and the shank (3 repetitions/point; SKL = skin lightness, SKA = skin yellowness, SKB = skin redness, SHL = shank lightness, SHA = shank yellowness, SHB = shank redness) using a Konica Minolta Chromameter CR-400 (Konica Minolta Inc., Osaka, Japan) [13].

### 2.3. Statistical Analysis

Data were analyzed using SAS 9.4 (SAS Institute Inc., Cary, NC, USA) statistic software. Analysis of variance was performed using the GLM procedure and the sources of variation considered were breed and breed * sex interaction. Student’s *t*-test was used to test significant differences between least square means [21]. Principal component analysis (PCA) was conducted separately on males and females with morphometric, colorimetric parameters and indexes as variables. Two different scatterplots were produced [22].

## 3. Results

The variance analysis results showed significant differences in morphometric and colorimetric parameters between breeds and sex within breeds, suggesting sexual dimorphism and key measures for characterizing BRZ and NIT Italian turkey breeds.

Mean values ± SE of the quantitative traits, LW, BL, KL, CC, WS, SL, SD, and SC, and indexes, including MASS, STOCK, and LLEG, are reported in Table 2. NIT was the heaviest breed (4.89 vs. 4.07 kg; *p* ≤ 0.05) and LW trait showed a clear sexual dimorphism with males (M) largely heavier than females (F) in both breeds (*p* ≤ 0.05). BL enhances the sexual dimorphism within each breed where toms’ and hens’ beak to tail lengths differ significantly, no difference between toms’ BL were recorded. KL was longer in the largest NIT breed: 15.08 vs. 13.75 cm (*p* ≤ 0.05). There were no significant differences in CC, WS, SL, SD, and SC between the two breeds; on the other hand, significant differences between the sexes were recorded (*p* ≤ 0.05). SC differences between toms and hens were not significant.

MASS was higher in NIT turkeys (8.26 vs. 7.0; *p* ≤ 0.05), STOCK was higher in BRZ birds (82.62 vs. 85.37; *p* ≤ 0.05), whereas no significant differences were recorded in the LLEG index.

The BCS value was 1 in all the scored turkeys.

Breeds’ colorimetric mean values ± SE are reported in Table 3. They reveal significant differences in skin lightness (L*) and redness (a*). NIT shows the highest values for L* and BRZ has the highest value for a* (*p* ≤ 0.05). In shank color, all the indexes showed a significant effect of the breed on their differences; lightness, redness, and yellowness were higher in BRZ compared with NIT.

**Figure 1:**
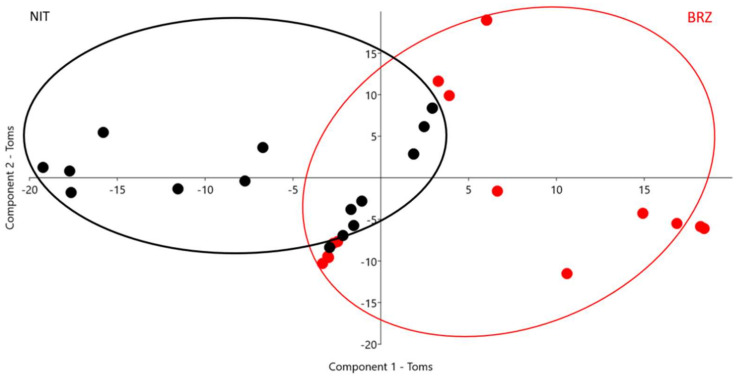
Scatter plot principal component analysis (PCA) for morphometric parameters and indexes in toms. Every spot represents a bird and every color a breed (BRZ = red: Brianzolo breed, NIT = black: Nero d’Italia breed; ellipses are a visual grouping sign).

**Figure 2:**
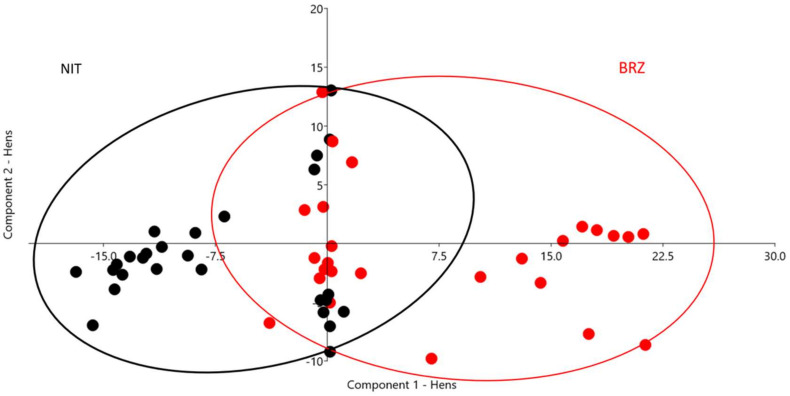
Scatter plot principal component analysis (PCA) for morphometric parameters and indexes in hens. Every spot represents a bird, and every color a breed (BRZ = red: Brianzolo breed, NIT = black: Nero d’Italia breed; ellipses are a visual grouping sign).

A PCA scatter plot for 17 parameters is presented in Figure 1 for toms and Figure 2 for hens. The first three components define more than 80% of the analyzed variance in males and females (80.49% toms; 82.11% hens).

PC1 determines 40.16% of the variance in males; the two determining variables are SHL (0.90) and SD (0.27). PC2 determines 28.90% of the variance, influenced by SD (0.66) and STOCK (0.49). The third component representing 11.42% of the variance is influenced by STOCK (0.58) and SD (0.53).

The scatter plot reveals a differentiation between males in the two breeds, mainly on PC1, where a reduced overlapping area is present.

PC1 determines 52.60% of the variance in females, and the two determining variables are SHL (0.94) and SHB (0.20). PC2 determines 17.14% and is influenced by SD (0.68) and WS (0.62). The three components representing 12.37% of the variance have the same influencing parameters, STOCK (0.84) and BL (0.46).

Clear differentiation between the hens of the two breeds is evident on Component 1, where reduced overlapping distributions are present.

In both sexes the birds set in the overlapping areas could be characterized by a lower differentiation in type defining traits, they could be considered a little bit ‘less typical’ than the birds clearly separating by breed on the two PC. PCA loadings and scree plots are reported in Supplementary Materials, Table S1 and Figure S1, respectively.

## 4. Discussion

NIT and BRZ are characterized by a very reduced registered population size [16]; our sample comprises all the breeders recorded in the conservational program, and they were housed in a controlled environment at the Poultry Unit, Animal Production Centre, University of Milan (Lodi, Italy). Significant differences in studied birds have been described for the considered parameters which showed their effectiveness in breeds differentiation. The first parameter described was LW. A routine weighing procedure and BCS were applied to evaluate farm animal nutritional status, body development, and general conditions [20]. LW plays a pivotal role in meat production species such as turkey. Sexual dimorphism was very evident in NIT and BRZ birds. Our results were determined according to the breeding goals described in Table 1. When we compared our breeds with other autochthonous breeds from different countries, we noticed that the average weight of local Tunisian turkey hens is 3.59 kg and toms’ average weight is 6.44 kg [11]. Nigerian turkeys appear to be very light, averaging 2.65 kg for females and 3.38 kg for the average males. Dalmatian turkey breeds from Croatia, part of the Austro-Hungarian Empire together with Veneto and Lombardy, are characterized by an average weight of 7.18 and 4.26 kg for toms and hens, respectively [23]. These weights are similar to NIT weights. The weight of toms for Mexican and Turkish turkeys ranges between 6.70 and 8.90 kg [12,23,24]. BL has limited differences in the considered breeds. Keel length is an important parameter that considers pectoral muscles to be the primary value point in turkey meat production: BRZ and NIT birds show significant differentiation [25]. In Tunisian turkeys, male and female KL ranges are 14.06–15.09 cm and 11.24–11.75 cm [11]. In general, KL shows smaller dimensional variations and is proportional to body size measurements [10]. Furthermore, Swatland [25] described that KL in meat and egg type turkeys was longer in pure meat-type birds from an early age. Heterosis and its positive effect on KL was recorded in some experimental lines in meat production [26]. Our KL results are dissimilar to those recorded by Oblakova et al. [18] in BUT hybrid toms, namely, 21.10 cm. SL and SC are important parameters that should be considered in defining selection plans for turkeys: the association between body weight and leg anatomy is fundamental in production and welfare optimization. Therefore, leg- and foot-related traits should be carefully evaluated in autochthonous turkey breeds because they objectively define indirect productive traits worth preserving as a genetic resource [26,27,28,29]. Shank length was similar in BRZ and NIT birds (10.57 and 10.85 cm); Dalmatian Turkeys, which are slightly heavier, have slightly higher SL values (15.77 cm males, 12.13 cm females) [23]. Leg disorders in meat-type poultry are related to reduced welfare and production quantity and quality [27]. In a recent study describing welfare issues in turkey production, Erasmus [29,30] lists leg and skeletal abnormalities among turkeys’ major welfare issues. The same conclusion was drawn by Robinson [29,30] about duck meat production. Locomotor ability is considered a key point in birds’ welfare in meat production and is assessed using gait score (GS) [27,29,30]. The effects of genetic selection on leg bones were described by Nestor et al. [26] who underline that leg bone measurements are less influenced by genetic variation between turkey lines compared with muscling measurements. BRZ and NIT birds are slow-growing turkeys traditionally reared in free-range conditions for their grazing ability in difficult terrains. Their anatomy may be due to adaptation through direct and indirect selection and high mobility levels [2,3]. The calculated indexes of MAS, STOCK, and LLEG reveal important information: NIT birds are slightly more massive than BRZ birds, which are slightly stockier. The two breeds are characterized by the same long-leggedness index, which underlines the breeds’ specific attitude toward locomotion and differentiation from meat-type turkeys [18]. BCS reveals the absence of overdeveloped pectoral muscles in these smaller heritage breeds. The visual appearance of poultry carcasses is a basic attribute in consumers’ choice orientation. Furthermore, genetics is one of the main factors affecting skin pigmentation together with diet formulation, health status, and processing methods during slaughter [14]. We recorded skin color in live birds fed the same diet to point out the genetic effects of the breed on skin pigmentation. NIT birds have a lighter, whitish color of skin. Shank color was significantly different between the two breeds. These results follow the standard definitions for breeds, which distinguish shank color as an important distinctive trait in standard turkey breeds (Table 1). NIT showed three indexes (lowest values) strictly related to darker shank pigmentation of the breeds. On the contrary, the lighter-colored shanks of BRZ birds were revealed by the higher values of L* a* b* indexes. A definition for the genetic basis of these described phenotypes would be helpful in classifying breeds [31].

The principal component analysis underlines the importance of morphometric and colorimetric parameters in differentiating turkey breeds. Although the historical origins and general type are similar, considering the high sexual dimorphism in these species and the significance of sexual dimorphism, two separate PCA were calculated by sex. Body size, proportions (STOCK, SD), and colorimetric parameters, particularly SHL and SHB, play pivotal roles in characterizing breeds. In toms, the two breeds clearly cluster on the first principal component where differentiation is based on shank traits: lightness and diameter; very few birds are set in the bordering area between the two breeds. Component two in toms focuses once more time the attention on shank diameter and stockiness which are higher in BRZ males. In hens, the results show similar tendency compared to those calculated in males: PC1 is strongly influenced by colorimetric indexes related to shank color evaluation (SHL and SHB). PC2 is influenced by shank diameter and wingspan. These results confirm the characterizing dark shank color and the more elongated silhouette of NIT compared to BRZ hens. In females, too, STOCK plays an important role in breed differentiation (PC3; 0.84) together with body length (PC3; 0.46).

## 5. Conclusions

The characterization of genetic resources is the first step towards their conservation and valorization not only under a biological but under an economical point of view. Furthermore, effectively characterized local breeds could better play a positive role in biodiversity conservation as breeds and as genetic reservoir for the highly productive species these birds belong to. For centuries, the analyzed breeds have been selected in poor, rural environments for their high coping ability, low-input demand, high brooding ability, and maternal behavior as brooders and foster mother hens. The phenotypes we measured are an expression of these selection goals and of the high variation present in native turkey breeds.

In particular, our investigation reveals stockier BRZ birds compared to slightly more elongated NIT birds under a morphometric point of view. The two breeds are strongly characterized by typical skin and shank coloration: NIT birds have very fair skinned with very dark, blackish shank, BRZ birds have a more colored skin and lighter pinkish shanks. In addition, high sexual dimorphism was defined in both NIT and BRZ birds an important aspect in local breeds which are considered more natural and ‘close to nature’ than commercial hybrids. Through morphological and colorimetric characterization selection target to differentiate NIT and BRZ birds could be defined.

Body weight and transversal diameters (STOCK) should be particularly considered in defining breeds’ differentiation traits. These parameters, relating to product quality, may be considered a starting point in selective programs planning. A characterized, effectively differentiated product, starting from recognizable live birds, could be more attractive for consumers, and so niche markets selling high-quality turkey meat may supply the economic resources needed to protect light, traditional, low-input turkey breeds [16]. Furthermore, their coping abilities in plein-air rearing systems strictly relate to their phenotypical traits. These characteristics should be considered in developing semi-extensive production facilities with large outdoor grazing areas in remote environments where intensive production systems cannot be applied. The objective data we produced by including productive traits and characteristic product information may be useful in upgrading breed standards too

Two different objectives should be reached: the morphological standardization of birds and the enhancement of genetic variability in these breeds at high risk of genetic erosion and extinction.

## Figures and Tables

**Table 1:** Breed description: name, Italian region of origin, status, feather color, shank color, and attitude (FIAV standard, [1]).

Breed	Brianzolo—BRZ	Nero d‘Italia—NIT
Region	Lombardy	Lombardy
Status	endangered population	rare breed
Weight M (kg)	4.0–4.6	4.0–6.0
Weight F (kg)	3.5–3.6	2.5–3.5
Feather Color	partridge brown	black
Skin Color	not standardized	not standardized
Shank Color	pinkish	dark red-violet-black
Attitudes	grazing ability, fast growth, resistance	resistance, brooding ability

**Table 2:** Quantitative traits: live weight (LW; kg), total body length (BL; cm), keel length (KL; cm), chest circumference (CC; cm), wingspan (WS; cm), shank length (SL; cm), shank diameter (SD; mm), shank circumference (SC; cm), massiveness (MASS; index), stockiness (STOCK), long-leggedness (LLEG; index) by breed and breed per sex interaction. Least-square means ± standard error.

Trait	BRZ			NIT			*p*	
	Population	Female	Male	Population	Female	Male	Breed	Breed * Sex
LW	4.07 ± 0.13	2.67 ± 0.16	5.47 ± 0.21	4.89 ± 0.12	3.02 ± 0.15	6.76 ± 0.20	≤0.05	≤0.05
BL	57.15 ± 0.60	51.4 ± 0.71	62.91 ± 0.96	58.44 ± 0.55	53.59 ± 0.66	63.31 ± 0.88	NS	≤0.05
KL	13.75 ± 0.39	12 ± 0.42	15.5 ± 0.67	15.08 ± 0.33	12.5 ± 0.38	17.66 ± 0.54	≤0.05	≤0.05
CC	47.32 ± 1.06	41.02 ± 1.30	53.62 ± 1.68	48.46 ± 1.01	41.93 ± 1.22	55 ± 1.62	NS	≤0.05
WS	54.15 ± 0.87	49.85 ± 1.05	58.45 ± 1.41	55.05 ± 0.81	49.71 ± 0.98	60.38 ± 1.3	NS	≤0.05
SL	10.57 ± 0.25	9.59 ± 0.31	11.56 ± 0.41	10.85 ± 0.23	9.63 ± 0.28	12.08 ± 0.38	NS	≤0.05
SD	9.28 ± 1.03	7.58 ± 1.23	10.98 ± 1.71	8.51 ± 0.95	6.7 ± 1.14	10.32 ± 1.53	NS	≤0.05
SC	2.78 ± 0.35	2.15 ± 0.42	3.42 ± 0.56	2.39 ± 0.32	1.82 ± 0.39	2.96 ± 0.52	NS	NS
MASS	7.0 ± 0.23	5.17 ± 0.28	8.83 ± 0.37	8.26 ± 0.21	5.76 ± 0.26	10.77 ± 0.34	≤0.05	≤0.05
STOCK	85.37 ± 1.04	80.15 ± 1.24	90.60 ± 1.67	82.62 ± 0.96	78.34 ± 1.15	86.90 ± 1.53	≤0.05	≤0.05
LLEG	17.90 ± 0.45	18.37 ± 0.54	17.44 ± 0.72	18.21 ± 0.41	17.77 ± 0.5	18.66 ± 0.67	NS	NS

BRZ = Brianzolo breed, NIT = Nero d’Italia breed.

**Table 3:** Colorimetric indexes: L* lightness (L), a* yellowness (A), b* redness (B): body skin (SK), and shank (SH). SKL: skin lightness; SKA: skin yellowness; SKB: skin redness; SHL: shank lightness; SHA: shank yellowness; SHB: shank redness by breed and breed per sex interaction. Least-square means ± standard error.

Trait	BRZ			NIT			*p*	
	Population	Female	Male	Population	Female	Male	Breed	Breed * Sex
SKL	65.35 ± 0.97	66.13 ± 1.12	64.58 ± 1.59	68.90 ± 0.95	69.02 ± 1.05	68.79 ± 1.56	≤0.05	NS
SKA	2.89 ± 0.48	2.08 ± 0.55	3.71 ± 0.78	0.67 ± 0.47	−041 ± 0.51	1.75 ± 0.78	≤0.05	≤0.05
SKB	2.14 ± 0.75	2.52 ± 0.86	1.77 ± 1.22	3.88 ± 0.73	5.14 ± 0.81	2.63 ± 1.22	NS	NS
SHL	63.98 ± 1.27	63.55 ± 1.46	64.41 ± 2.06	39.08 ± 1.23	38.98 ± 1.36	39.20 ± 2.07	≤0.05	NS
SHA	2.37 ± 0.48	2.01 ± 0.56	2.73 ± 0.79	−0.89 ± 0.475	−0.76 ± 0.52	−1.03 ± 0.79	≤0.05	NS
SHB	11.34 ± 0.38	11.26 ± 0.44	11.06 ± 0.62	5.62 ± 0.37	6.04 ± 0.41	5.19 ± 0.62	≤0.05	NS

BRZ = Brianzolo breed, NIT = Nero d’Italia breed.

## Data Availability

Not applicable.

## Supplementary Materials

The following supporting information can be downloaded at: https://www.mdpi.com/article/10.3390/ani12050571/s1. Figure S1: PCA: Scree plot. Eigenvalues (%): Explained Variance (%), Table S1: PCA: Loadings.
