# Supplementary material for: Morphological Characterization of Two Light Italian Turkey Breeds
Source: Animals (Basel). 2022 Feb 24;12(5):571. doi: 10.3390/ani12050571 (PMC8909484; doi:10.3390/ani12050571)

Table S1 PCA: Loadings

	TOMS			HENS		
	**PC 1**	**PC 2**	**PC 3**	**PC 1**	**PC 2**	**PC 3**
**BW**	0.001864	0.054638	0.11727	-0.01157	0.029013	-0.02217
**SL**	0.016896	0.042594	0.11008	0.009183	0.004757	-0.04466
**BL**	0.046375	0.080455	0.012363	-6.12E-02	0.076	-0.45856
**KL**	-0.01565	0.004017	-0.00249	-0.00388	-0.00244	0.003616
**BrC**	0.099572	0.38339	-0.35003	-0.00757	0.15832	0.093798
**SC**	0.10434	0.23142	0.15797	0.024623	0.24403	-0.05768
**SD**	0.268	0.66194	0.52831	0.038407	0.68272	-0.13467
**WS**	-0.06933	-0.05185	0.38343	-0.07355	6.19E-01	-2.50E-02
**MASS**	0.002353	0.064359	0.17562	-0.01897	0.039464	0.004063
**STOCK**	0.091975	0.49179	-0.57598	0.082399	0.19674	0.84292
**LLEGG**	-0.00376	0.054343	0.17095	0.017794	-0.02647	0.085662
**SKL**	-0.14225	0.034963	0.008738	-0.14693	-1.20E-01	1.45E-01
**SKA**	0.090763	0.000791	0.042876	0.097496	2.89E-02	-6.24E-02
**SKB**	-0.02396	0.010279	-0.01842	-0.06534	0.011851	0.068462
**SHL**	0.90082	-0.30128	-0.05306	0.94369	-0.01745	-0.05903
**SHA**	0.13691	-0.01966	0.0428	0.12012	0.0507	-0.07158
**SHB**	0.17929	-0.07669	-0.04055	0.20166	-0.02428	0.014143

Figure S1 PCA: Scree plot. Eigenvalues (%): Explained Variance (%)

A - TOMS

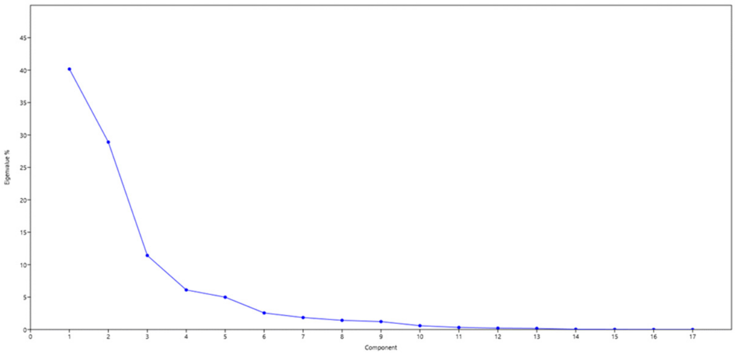

B – HENS

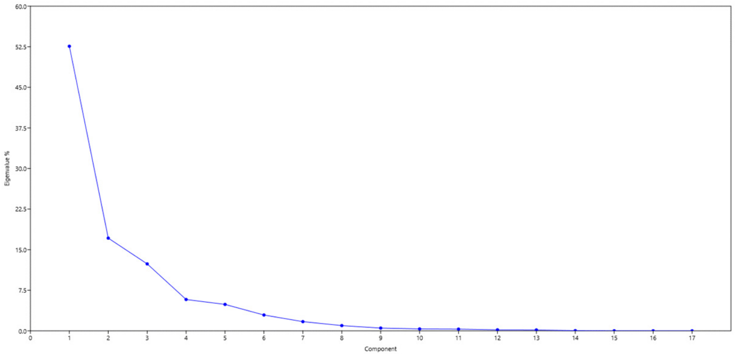

Supplement: Supplementary file 1 [file animals-12-00571-s001.zip › animals-1525382-supplementary.pdf]
